# Functional Analysis of Differentially Expressed Acetylated Spermatozoal Proteins in Infertile Men with Unilateral and Bilateral Varicocele

**DOI:** 10.3390/ijms21093155

**Published:** 2020-04-30

**Authors:** Manesh Kumar Panner Selvam, Luna Samanta, Ashok Agarwal

**Affiliations:** 1 American Center for Reproductive Medicine, Cleveland Clinic, Cleveland, OH 44195, USA; pannerm@ccf.org (M.K.P.S.); lsamanta@ravenshawuniversity.ac.in (L.S.); 2 Redox Biology Laboratory, Department of Zoology, Ravenshaw University, Cuttack 753003, India

**Keywords:** sperm proteome, acetylation, bioinformatics, varicocele, male infertility

## Abstract

Sperm proteins undergo post-translational modifications, such as phosphorylation, acetylation, and ubiquitination, which in turn play a key role in determining their fertilizing ability. In the current study, we examined the sperm proteome of men with unilateral and bilateral varicocele to identify the key proteins affected by acetylation to gain an insight into the difference in the severity of affected sperm function in the latter. An LTQ-Orbitrap Elite hybrid mass spectrometer system was used to profile the sperm proteome in pooled unilateral and bilateral varicocele patients. Bioinformatics database and tools, such as UniProtKB, Ingenuity Pathway Analysis Software (IPA) and Metacore, were used to identify the differentially expressed proteins (DEPs) involved in the acetylation process. A total of 135 DEPs in the spermatozoa of unilateral and bilateral varicocele patients were found to be affected by acetylation. The majority of these DEPs found were regulated by key transcription factors such as androgen receptor, p53, and NRF2. Furthermore, the DEPs predicted to be affected by the acetylation process were associated with fertilization, acrosome reaction, mitochondrial dysfunction and oxidative stress. Aberrant expression of proteins and their differential acetylation process may affect the normal physiological functions of spermatozoa. Protein–protein interactions identified dysregulation of the proteasome complex in the bilateral varicocele group. Damage to the proteasome complex may result in aggregation of the misfolded proteins, which in turn increase sperm DNA damage and apoptosis in patients with bilateral varicocele.

## 1. Introduction

The incidence of infertility is about 9% of the total population, and around 20% of cases are contributed only by the male factor [1]. Varicocele is one of the most relevant problems related to male infertility, with a prevalence of 15% of the male population [2] and accounting for 40% to 80% of primary and secondary male factor infertility, respectively [3,4]. The occurrence of unilateral varicocele is more common compared to bilateral varicocele [5]. Poor semen quality with increased seminal oxidative stress and sperm DNA fragmentation (SDF) has been frequently encountered in infertile men with varicocele [6].

Since proteins are considered the functional molecules of the cell, alteration in their expression has deleterious effects on sperm functions. In the post-genomic era, the global proteome is widely used to identify alterations in proteins that can be associated with the etiology of infertility. Although there are only a few studies focused on proteomics/proteins expression and varicocele, it has already been identified that some proteins related to sperm function are altered [7,8,9]. Agarwal et al. examined the proteome of sperm in unilateral and bilateral varicocele samples using liquid chromatography-mass spectrometry (LC-MS) [10]. Key proteins, such as PKAR1A, CCT6B, AK7, TEKT3, TCP11, HSPA2, and ODF2, involved in the stress response, sperm function, and mitochondrial dysfunction were validated and proposed as potential biomarkers for infertile men with unilateral or bilateral varicocele [10]. Besides altered expression, many proteins undergo post-translational modification (PTM), thereby experiencing either a loss their functions or hyperactivation. Either case may impair the cellular function.

Since spermatozoa are transcriptionally and translationally silent [11], the role of PTM in regulating post-testicular maturation and post-ejaculation events cannot be ruled out. Acetylation, a prominent modification of proteins reported in eukaryotic cells, involves the transfer of an acetyl group from acetyl CoA to a free amino group of a target protein [12]. In human spermatozoa, acetylation mainly mediates nuclear histone modification during spermatogenesis [13], with a positive correlation between the acetylation of histones and SDF [12]. Apart from histones, certain important proteins related to sperm function, such as ODF2, TEKT3, GAPDHS, GPX, CABYR, and ACRV1, are acetylated in human sperm [14]. Furthermore, several other acetylated proteins are also involved in cellular signal transduction and the regulation of different biochemical activities [15]. The in-depth analysis by Yu et al. revealed 973 lysine-acetylated sites matching with 456 human sperm proteins, and 671 novel lysine acetylation sites were predicted matching with 205 novel lysine-acetylated proteins [12]. The three conserved motifs (XXXKYXXX, XXXKFXXX, and XXXKHXXX) exhibited by these proteins were annotated to function in multiple metabolic processes, and were localized predominantly in the mitochondrion and cytoplasmic fractions. However, these authors used capacitated and non-capacitated spermatozoa to validate the role of protein acetylation in sperm function. Previous studies by our group demonstrated that the majority of differentially expressed proteins in varicocele patients compared to fertile donors are predominantly of mitochondrial origin and involved in various metabolic functions, including the stress response [10,16]. Similarly, when the proteome profile of unilateral varicocele to bilateral varicocele was compared, ~122 proteins were predicted to have acetylation sites while the principal pathways were predicted to be protein degradation, free radical scavenging, PTMs, mitochondrial function, and apoptosis [16]. Therefore, in the present study, we performed a secondary analysis of the previously obtained sperm proteomic data to identify the proteins susceptible to acetylation and their role in various sperm functions in unilateral and bilateral varicocele patient samples in order to understand the mechanism(s) involved with disease severity in bilateral varicocele.

## 2. Results

### 2.1. Semen Analysis and Advanced Sperm Function Tests

Sperm concentration, motility, and normal morphology in both the varicocele group were not significantly different. The seminal reactive oxygen species level was more than the cut-off value of 93 RLU/s/10^6^ sperm whereas the SDF levels was higher in the bilateral varicocele group (Supplementary Table S1).

### 2.2. Proteomic Analysis

**Figure 1:**
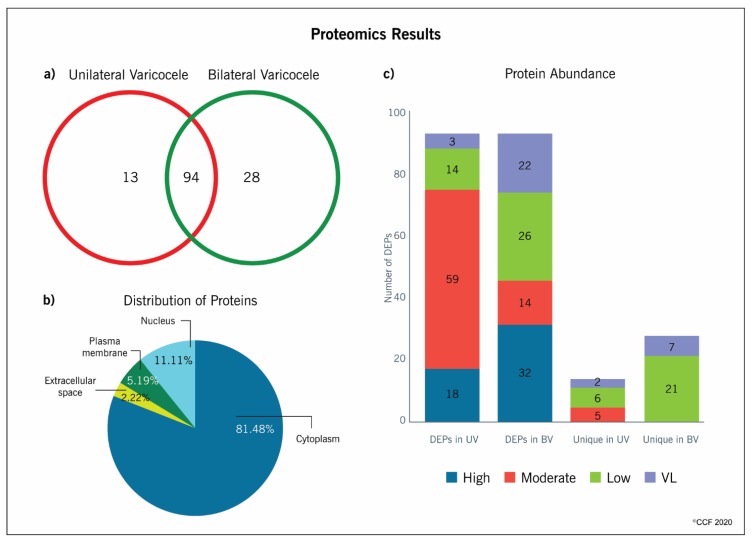
(**a**) Differential expression proteins predicted to be acetylated in unilateral and bilateral varicocele, (**b**) Abundance of differentially expressed proteins, (**c**) Distribution pattern of differentially expressed proteins involved in the acetylation process.

Reanalysis of the global proteomics data of spermatozoa from unilateral and bilateral varicocele based on the normalized spectral abundance factor (NSAF) ratio revealed significant differential expression of 135 proteins having susceptible sites for acetylation. Among these, 94 DEPs (62 overexpressed and 32 underexpressed in unilateral varicocele) were common to both groups whereas 13 and 28 proteins were unique to the unilateral and bilateral varicocele group, respectively (Figure 1a). Spectral counts of these DEPs indicated that a majority were moderately abundant (*n* = 59) and highly abundant (*n* = 18) in the spermatozoa of men with unilateral varicocele, while in bilateral varicocele, 32 DEPs were highly abundant, but maximum DEPs were either lowly (*n* = 26) or very lowly (*n* = 22) abundant in spermatozoa (Figure 1b). Most of these uniquely expressed proteins in both the unilateral and bilateral varicocele groups were present in low and very low abundance levels (Figure 1b).

All the DEPs overexpressed in the spermatozoa of men with bilateral varicocele compared to underexpressed proteins in unilateral varicocele were present in a high abundance except for AKR1B1 and PSMD13 (Table 1). The underexpressed proteins in the bilateral varicocele group compared to overexpressed proteins in unilateral varicocele were either low or very low and moderately abundant except for ENO1 and MDH2, which were highly abundant (Table 2). Certain uniquely expressed proteins in the unilateral group (CAT, PNP, ACCAA2, SELENBP1) were present in moderate abundance. However, the other proteins unique to the bilateral group were either low or very low in abundance (Table 3).

### 2.3. Distribution Pattern and Functional Annotation of Proteins

In the current study, GO enrichment analysis and Ingenuity Pathway Analysis (IPA) annotation identified that 81.48% and 11.11% of the proteins involved in the acetylation function were present in the cytoplasm and in the nucleus of spermatozoa, respectively (Figure 1c). Functional analysis using advanced databases, such as GO Term Finder, UniProt, and database for annotation, visualization and integrated discovery (DAVID), revealed that a maximum number of proteins involved in the acetylation function had multifunctional properties. Further, annotation analysis revealed that these DEPs were associated with apoptosis or DNA damage, energy metabolism or stress regulation, mitochondrial dysfunction, and sperm function (Supplementary Table S2).

### 2.4. Classification of DEPs with Predicted Acetylation

All overexpressed DEPs in the bilateral varicocele group were associated with DNA damage, apoptosis, or oxidative stress mechanisms. Moreover, important proteins related to spermatogenesis, sperm function, and mitochondrial dysfunction, such as CLGN, PARK7, HSPA9, HIST1H2BA, DECR1, DLAT, GSR, SDHA, PPP3CA, and SUCLA2, were underexpressed (Supplementary Table S2). Some of the proteins that were highly abundant in the spermatozoa of men diagnosed with both unilateral and bilateral varicocele included ENO1, ACTB, EEF1A1, FASN, HSPA5, HSP90AA1, MYH9, PKM, TUBA3C, and TUBB4B. Nonetheless, proteins, such as CLGN, FTH1, MDH1, MIF, PPP3CA, SUCLA2, and PSMA7, involved in sperm function, apoptosis, and oxidative stress were present in a low and very low abundance in the unilateral and bilateral varicocele group, respectively (Supplementary Table S2).

### 2.5. Identification of DEPs Involved in Important Networks

**Figure 2:**
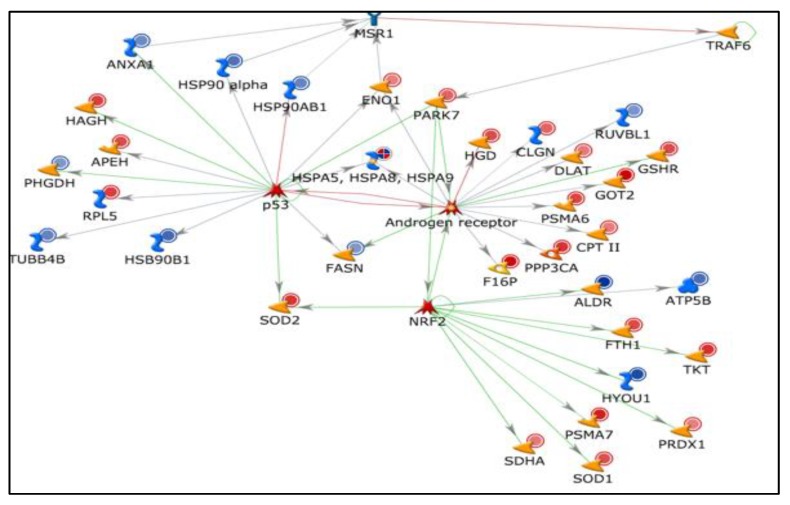
Transcription factors androgen receptor, p53, and NRF2 affected by the differential expression of proteins involved in the acetylation process.

**Figure 3:**
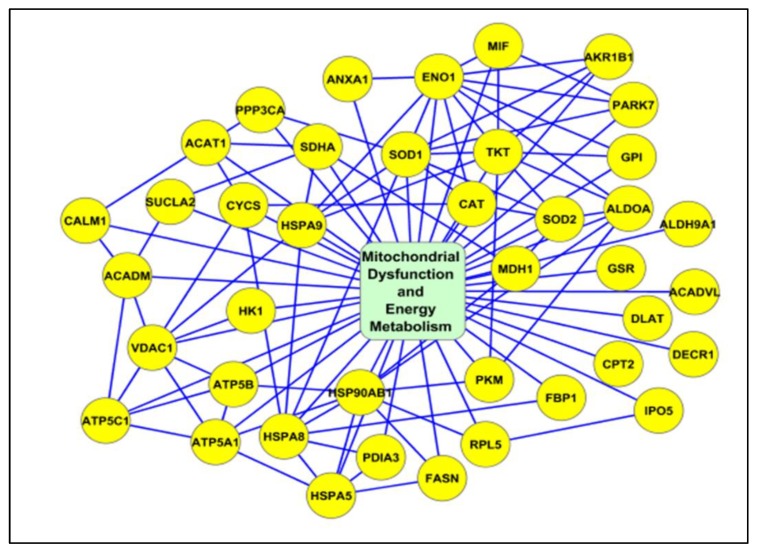
Interaction network of acetylated proteins associated with mitochondrial dysfunction and energy metabolism.

**Figure 4:**
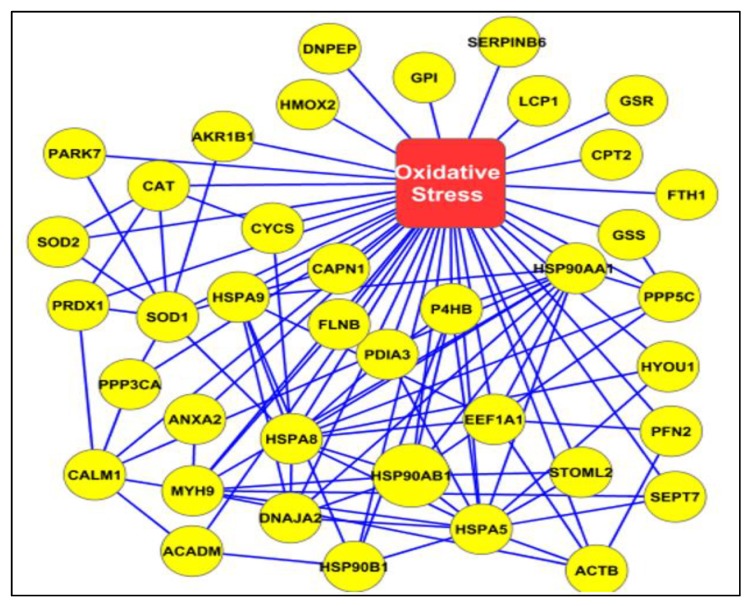
Interaction network of acetylated proteins associated with oxidative stress.

**Figure 5:**
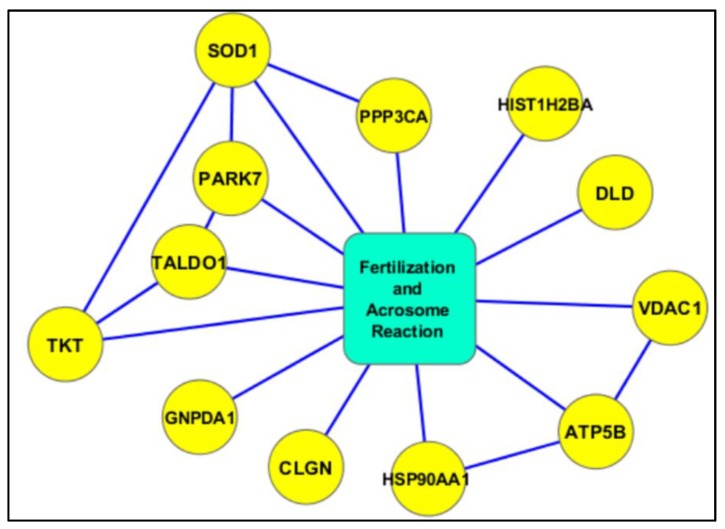
Interaction network of acetylated proteins associated with fertilization and the acrosome reaction.

Metacore analysis identified that the transcription factors of androgen receptor, p53, and NRF2 are the main proteins affected by the differential expression of proteins with potential acetylation sites (Figure 2). Networks developed using DEPs based on their biological function demonstrated that a majority of proteins were associated with mitochondrial dysfunction and energy metabolism (Figure 3), and oxidative stress (Figure 4). We also noticed proteins were involved in fertilization and the acrosome reaction (Figure 5).

### 2.6. Protein–Protein Interaction

**Figure 6:**
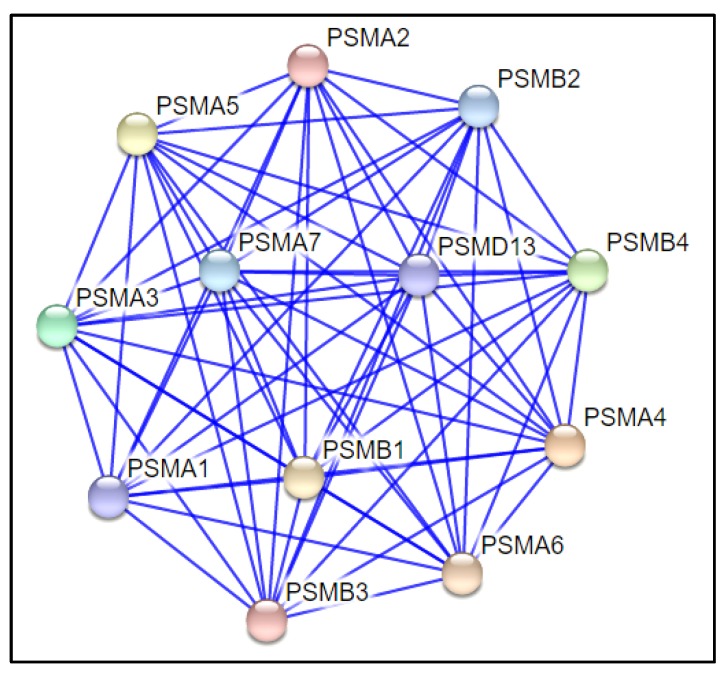
STRING analysis showing the interaction among the proteins involved in the proteasome complex.

Further search tool for the retrieval of interacting genes/proteins (STRING) analysis for all DEPs involved in the acetylation process demonstrated key protein–protein interactions in the varicocele condition. These DEPs were involved in regulating sperm functions. Interestingly, the majority of the proteins associated with the proteasome complex were involved in the acetylation process. STRING analysis displayed the DEPs (PSMA1, PSMB3, PSMA6, PSMA4, PSMB4, PSMB2, PSMA2, PSMA5, PSMA3, PSMA7, PSMD13, and PSMB1) present in the proteasome complex by interaction with each other through a binding process (Figure 6).

### 2.7. Potential Protein Biomarkers and Western Blot Analysis

**Figure 7:**
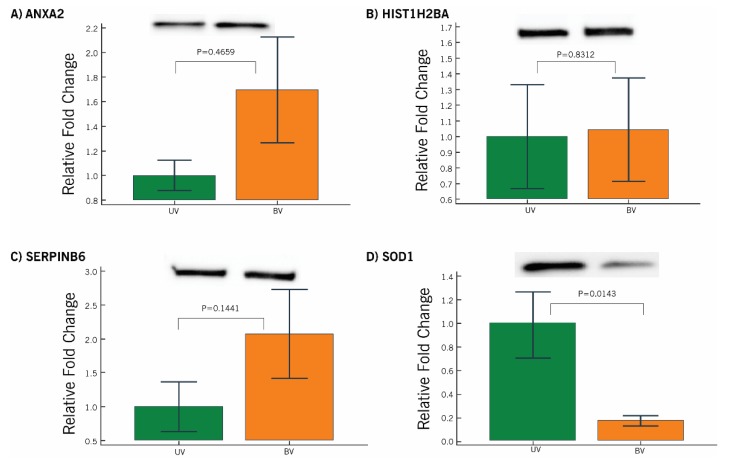
Western blot analysis of proteins associated with the acetylation process in unilateral and bilateral varicocele infertile men. (**A**) Annexin A2 (ANXA2), (**B**) histone H2B type 1-A (HIST1H2BA), (**C**) serpin B6 (SERPINB6), (**D**) superoxide dismutase 1 (SOD1). Results are expressed as mean ± standard error of mean and in fold variation to the unilateral varicocele group.

Functional proteins related to the acetylation process involved in the acrosome reaction and fertilization process, mitochondrial dysfunction, and oxidative stress were listed as potential protein biomarkers to identify PTM defects pertaining to the acetylation process (Table 4). The presence of acetylated proteins (ANXA2, HIST1H2BA, SERPINB6, and SOD1) was demonstrated in acetyl-lysine immunoprecipitated proteins. Further, western blot (WB) analysis revealed SOD1 was significantly underexpressed (*p* = 0.0143) in the bilateral varicocele group (Figure 7).

## 3. Discussion

PTMs of sperm proteins are necessary for the normal physiological functions of spermatozoa [17]. Proteomic analysis of sperm proteins was able to detect the maximum number of proteins present in the spermatozoa. Bioinformatic analysis revealed the involvement of these proteins in specific biological, molecular, and cellular pathways. Furthermore, using a bioinformatics approach, sperm proteins involved in important PTMs, such as phosphorylation, methylation, and acetylation, were also predicted. Since acetylation occurs on numerous and diverse proteins and affects many protein functions, such as DNA binding, enzymatic activity, protein stability, protein–protein interaction, and peptide-receptor recognition, we carried out a detailed analysis of DEPs with the potential of acetylation.

Previous studies used enrichment steps to isolate sperm proteins involved in PTMs, such as phosphorylation and acetylation [12,14]. Further, the LC-MS/MS platform was used to identify proteins and peptides. However, it was performed using physiologically normal spermatozoa [12]. Acetylated proteins in the spermatozoa are involved in sperm functions, such as energy metabolism, capacitation, acrosome reaction, sperm–egg recognition, and fusion [12,14]. The fertilization potential of the spermatozoa is compromised in men with varicocele-mediated male infertility. Until now, no studies have investigated the role of acetylation proteins in infertile men with unilateral or bilateral varicocele. In the current study, we used a global proteomic and bioinformatic approach to identify proteins susceptible to acetylation and further validated the presence of key acetylated proteins in sperm from men with unilateral and bilateral varicocele to understand the etiologies of sperm dysfunction in both cases.

In general, the concentration or abundance of proteins with PTMs in spermatozoa are comparatively lower than that of proteins in their native form. More than 1000 lysine acetylation sites were reported in the proteins of capacitated human sperm and were involved in sperm functions, such as sperm motility and the fertilization process [14]. In the current study, we also observed that most of the proteins predicted to be acetylated were present in a low or very low abundance in both groups (Figure 1b). In the human spermatozoa, the majority of these proteins were reported to be acetylated and involved in sperm motility, capacitation, sperm–egg recognition, fusion of sperm–egg plasma, and fertilization [12]. Acetylation is a dynamic process that regulates cellular function, and optimal spatio-temporal acetylation is required for proper functioning of the cell. Even a slight dysregulation in the expression of proteins may affect the acrosome reaction and fertilization potential of the spermatozoa [14].

Proteomic studies in varicocele patients reported aberrant expression of proteins involved in capacitation, acrosome reaction, and fusion of the sperm with the zona-pellucida [10,16]. In the present study, IPA analysis revealed that the identified proteins that carry out acetylation were proteins related to the acrosome reaction and fertilization. Hyperacetylation of histones is an important event during spermatogenesis [15]. Further, histone acetylation facilitates its replacement with protamines for denser packing of the sperm DNA [15]. Our bioinformatics analysis revealed that the differential expression of HIST1H2B is involved in the acetylation process, in both unilateral and bilateral varicocele patients. Acetylation of HIST1H2B in varicocele patients (unilateral and bilateral) was confirmed by WB analysis. Hence, the expressional change in the acetylated HIST1H2B may have a direct impact on the quality of sperm chromatin in the ejaculated spermatozoa. Furthermore, aberrant expression of acetylated HIST1H2B may serve as a marker for sperm chromatin integrity in varicocele patients.

In the current study, the majority of proteins involved in the acetylation process were under the regulation of transcription factors, androgen receptor, p53, and NRF2. The androgen receptor plays an important role in regulating the action of testosterone, which is required for spermatogenesis [18,19]. Further, the expression of proteins involved in oxidative stress, apoptosis, and cell signaling is affected due to the presence of defective androgen receptors [20]. Their presence in human spermatozoa was demonstrated by WB and immunocytochemistry [21], and on the basis of the androgen concentration, they have the ability to modulate the PI3K/AKT pathway to regulate apoptosis in spermatozoa [22]. In varicocele patients, malfunctioning of the androgen receptor may be due to decreased testosterone levels [23]. Androgen receptors are also affected by PTMs, where acetylation specifically induces apoptosis [24,25]. From the network created, it is clear that the majority of the proteins involved in the acetylation process under the regulation of the androgen receptor are underexpressed in patients with bilateral varicocele (Figure 2). This indicates that the damage to spermatogenesis is higher in bilateral varicocele patients as compared to patients with unilateral varicocele.

The transcription factor p53 is involved in the regulation of apoptosis and is reported as a marker of sperm DNA damage [26,27,28]. Augmented expression of p53 along with PARP and Bak was reported in the spermatozoa of varicocele patients, implying a higher incidence of apoptosis in these patients in comparison with normal individuals [29]. In the current study, we noticed that p53 was involved in the regulation of the DEPs associated with the acetylation process. It is not out of context to mention here that intracellular ANXA2 interacts with p53-mediated apoptosis [30]. WB analysis of acetylated ANXA2 further endorsed the role of acetylation by inducing more apoptotic sperm death in bilateral varicocele. In fact, a lower expression of ANXA2 in human sperm is correlated with high levels of SDF [31]. Further, N-terminal acetylation of membrane-bound ANXA2 is required for S100A10 binding for an enhancement of its activity [32]. Thus, the aberrant expression of proteins involved in the acetylation process may trigger p53 transcription factor, which, in turn, activates the apoptosis process in the defective spermatozoa of varicocele patients.

The transcription factor NRF2 regulates the expression of antioxidant enzymes by activation of the antioxidant responsive element (ARE) [33] and was associated with acetylated sperm proteins [34]. In varicocele patients, NRF2 was linked with mitochondrial dysfunction [35]. Validation of the downstream effecter molecule of NRF2, i.e., antioxidant enzyme Cu/Zn-superoxide dismutase (SOD1), further supports the computational findings. WB analysis of immunoprecipitated SOD1 protein present in the network confirms that the proteins identified as acetylated proteins undergo PTM in the spermatozoa of varicocele patients. In fact, the acetylation of lysine residues near the active site has also been implicated in the inhibition of manganese superoxide dismutase (SOD2) while oxidative stress stimulates SIRT3 to deacetylate SOD2, leading to SOD2 activation and reactive oxygen species (ROS) reduction [36,37,38]. Therefore, the differential acetylation of SOD1 in both groups might be due to differences in the incidence of oxidative stress as both the testes in bilateral varicocele experience hypoxia, while in unilateral varicocele, the contra-lateral testis has normal blood flow.

In general, mitochondrial dysfunction and oxidative stress are the main factors affecting the quality of spermatozoa in varicocele pathology. Earlier sperm proteomic studies in varicocele patients revealed mitochondrial dysfunction and underexpression of mitochondrial proteins [10,35]. In the current study, network analysis identified the acetylation of important sperm proteins, such as PRDX1 and SDHA, associated with oxidative stress and mitochondrial dysfunction, respectively (Figure 3 and Figure 4).

Peroxiredoxins play a major role in the regulation of oxidative stress by modulating ROS levels [39]. Cui et al. reported an increased expression of oxidative stress response protein PRDX1 in immature sperm [40]. Acetylation of the PRDX1 protein increases its reducing activity [41]. Our proteomic data revealed that PRDX1 was overexpressed in unilateral varicocele compared to bilateral varicocele. This may be due to the compensatory redox mechanism being activated in the unilateral varicocele condition [42]; alternatively, lower expression of this protein with low levels of acetylation may be responsible for the higher damage in bilateral varicocele patients. In fact, bilateral varicocele patients in general present with severe anomalies in sperm parameters. Hence, the acetylation state of PRDX1 is critical in varicocele patients with oxidative stress-mediated infertility. Unlike PRDX1, SDHA is a mitochondrial protein and a subunit of the succinate dehydrogenase complex that is involved in the tricarboxylic acid cycle (TCA) cycle, electron transport chain, and energy metabolism. The expression of SDHA was dysregulated in asthenozoospermic patients [43]. Hyperacetylation of SDHA decreases its electron-transferring activity [44,45]. In unilateral varicocele patients, we observed the overexpression of SDHA protein in comparison to bilateral varicocele, further validating more functional impairment of spermatozoa in the bilateral varicocele group. Further, WB analysis of the immunoprecipitated proteins ANXA2 and SERPINB6 involved in the same network confirmed the proteins associated with the oxidative stress response are acetylated in the spermatozoa of varicocele patients.

In the current study, seven alpha-type subunits (PSMA1, PSMA2, PSMA3, PSMA4, PSMA5, PSMA6, and PSMA7) and four beta-type subunits (PSMB1, PSMB2, PSMB3, and PSMB4) of the core 20S proteasome were predicted to be potential sites for acetylation and were overexpressed in unilateral varicocele compared to bilateral. Here, 20S core proteasome is the catalytic site responsible for maintenance of protein homeostasis by removing misfolded or damaged proteins that could impair cellular functions, and by removing proteins whose functions are no longer required. Furthermore, lysine acetylation of 20S cardiac proteasome has been reported to augment its activity [46]. It is not out of context to mention here that proteasomes are located in the mammalian sperm acrosome and on the acrosomal surface. Protein kinase A modulates the enzymatic activity of the proteasome during a progesterone-induced acrosome reaction [47]. Furthermore, after capacitation, the acrosomal proteasomes facilitate the degradation of zona pellucida glycoproteins, leading to fertilization. In a recent study, Zigo et al. (2019) showed that both parallel and sequential treatments of ejaculated and capacitated spermatozoa in the absence and presence of a proteasome inhibitor demonstrated putative sperm proteasome-associated sperm proteins in a compartment-specific manner. The investigators further demonstrated that other proteins associated with capacitation, such as P47/lactadherin, ACRBP, ADAM5, and SPINK2, were processed by the proteasome, and proteasomal inhibitors slowed down capacitation-induced reorganization of the outer acrosomal membrane [48]. Therefore, a decline in the expression of these core subunits of 20S proteasome in bilateral varicocele will decrease the chance of fertilization in the spermatozoa of these patients.

## 4. Conclusions

In this study, we identified sperm DEPs that are able to regulate the protein acetylation process in infertile men with unilateral and bilateral varicocele. Aberrant expression of acetylated proteins associated with oxidative stress and mitochondrial dysfunction affects the fertilization potential of sperm in varicocele patients. In addition, the acetylation status of proteins, such as HIST1H2B, PRDX1, SDHA, and SOD1, can serve as a biomarker for PTM defects pertaining to the acetylation process in the ejaculated spermatozoa of varicocele patients.

## 5. Materials and Methods

### 5.1. Patient Characteristics

This study was approved by the Institutional Review Board (IRB) of the Cleveland Clinic (IRB # 17-422), Cleveland, OH, United States. All participants signed an informed written consent form at the Andrology Center, Cleveland Clinic. Semen samples were obtained from 50 varicocele patients (33 unilateral and 17 bilateral). The majority of the varicocele patients were diagnosed with grade 1 or 2 (unilateral = 83.6% and bilateral = 76.4%) (Supplementary Table S1).

### 5.2. Proteomic Analysis

Proteomic raw data from our previous studies on unilateral [16] and bilateral [10] varicocele were reanalyzed. Experimental details of the global proteome analysis, including sample preparation and LC-MS/MS data analysis, are described in detail in our previous publication [10]. In the present study, we analyzed the functional proteins having potential acetylation sites.

### 5.3. Database Searching and Protein Identification

Tandem mass spectra were extracted by Proteome Discoverer version 1.4.1.288. Charge state deconvolution and deisotoping were not performed. All MS/MS raw files were analyzed using Mascot (Matrix Science, London, UK; version 2.3.02), Sequest (Thermo Fisher Scientific, San Jose, CA, USA; version 1.4.0.288), and X! Tandem (The GPM, thegpm.org; version CYCLONE (2010.12.01.1). Mascot, Sequest, and X!Tandem were set up to search the human reference with a database (33,292 entries) assuming trypsin as the digestion enzyme. The mass tolerance for the parent ion was set to 10 parts per million (PPM) and for a fragment ion with 1.0 Da.

Scaffold (version 4.0.6.1; Proteome Software Inc., Portland, OR) was used to validate MS/MS-based peptide and protein identifications. Peptide and protein identifications were accepted as previously described [10]. Protein probabilities were assigned by the Protein Prophet (Systems Biology, Seattle, WA, USA) algorithm. Protein annotation was performed using Gene Ontology (GO) terms from National Center for Biotechnology Information (NCBI).

### 5.4. Quantitative Proteomics

In general, the relative quantity of the proteins was determined by comparing the number of spectra, termed spectral counts (SpCs), used to identify each protein. The total number of mass spectra (SpCs) that matched peptides to a particular protein was used to measure the abundance of proteins in the complex mixture. To overcome the sample-to-sample variation associated with replicate analyses of a sample and the fact that longer proteins tend to have more peptide identifications than shorter proteins, the NSAF approach was applied prior to relative protein quantification. The criteria used to identify and categorize DEPs based on the SpC were explained in our earlier publication [10].

### 5.5. Large-Scale Identification of Acetylation Sites

The carbamido methylation of cysteine was set as a fixed modification. For the identification of potential PTM sites, protein N-terminal diglycine/acetylation, diglycine/acetylation of lysine, and oxidation of methionine and pyro-glutamination for N-terminal glutamine were set as variable modifications to identify the ubiquitination and acetylation sites in our database search. A maximum of three or six missed cleavages were allowed, setting Trypsin as the proteolytic enzyme. The mass tolerance was set to 3 parts per million (ppm) for peptide masses and 0.8 Da for MS/MS peaks, respectively. In the process of peptide identification, we conducted a decoy database search by Mascot, and applied a filter to satisfy a false positive rate lower than 1%.

Flanking amino acid sequence analysis: For the representation of position weight matrices (PWMs) for (−9) to (+9) amino acid residues surrounding all of the identified lysine ubiquitination and acetylation sites, the probability of the observed amino acid residues, at each position on the flanking sequences of the lysine modification sites, was normalized by the abundance ratio of each amino acid in the RefSeq human protein database. Visualization of the statistically extracted sequence motifs, based on our large-scale lysine modification proteome data, was also performed using motif-X [49,50].

Pathway analysis: The computational analysis for the statistical extraction of canonical pathways was performed using IPA (QIAGEN, Redwood City, CA, USA) [51]. The proteins modified with the ubiquitination and/or acetylation were uploaded into the IPA software (version 2018-2019), and the top canonical pathways associated with the uploaded proteins were listed along with the *p*-values calculated using a right-tailed Fisher’s exact test.

### 5.6. Functional Analysis

Functional annotation and enrichment analysis were performed using publicly available bioinformatic annotation tools and databases, such as GO Term Finder, GO Term Mapper, UniProt, Software for researching annotations of proteins (STRAP), and DAVID (http://david.niaid.nih.gov). Proprietary software packages, such as IPA from Ingenuity^®^ Systems, were also used to obtain a consensus-based comprehensive functional context for the list of proteins involved in the acetylation process derived from the proteome.

### 5.7. Bioinformatic Analysis of Protein–Protein Interaction

STRING was used to display the functional and physical link between the proteins based on the criteria: Neighborhood, gene fusion, occurrence, co-expression, experimental evidences, existing databases, and text mining. This includes all proteins with potential acetylation sites with respect to spermatogenesis, spermatid differentiation or development, sperm motility, capacitation, acrosome reaction, mitochondrial dysfunction, energy metabolism, apoptosis, DNA damage, DNA methylation, and oxidative stress.

### 5.8. Immunoprecipitation and Western Blotting of Acetylated Proteins

Acetylation of sperm proteins in both unilateral (*n* = 6) and bilateral varicocele patients (*n* = 6) was demonstrated using WB. Immunoprecipitation of acetylated proteins was carried out using anti-acetyl Lysine antibody (ab190479, Abcam, USA) followed by WB detection of selected acetylated proteins. The criteria applied for the selection of DEPs involved in the acetylation process were as follows: (i) Proteins involved in the networks; (ii) abundance of the protein must be moderate or high in any one group; and (iii) proteins with a well-described function in the literature. Four proteins (ANXA2, HIST1H2BA, SERPINB6, and SOD1) were chosen for validation by WB in both the unilateral and bilateral varicocele group.

Immunoprecipitated acetylated proteins were first loaded into a 4–15% SDS–PAGE for 2 h at 90 V. The resolved proteins were transferred onto polyvinylidene difluoride (PVDF) membranes and analyzed as described earlier [52]. The expression levels of the WB-validated proteins were normalized against the global acetylated proteins (Supplementary Figure S1) and compared between unilateral and bilateral varicocele using the Mann–Whitney test and *p* < 0.05 was considered significant. Data analysis was performed using MedCalc Statistical Software (version 17.8; MedCalc Software, Ostend, Belgium).

## Figures and Tables

**Table 1:** Comparison of the enrichment of lysine-acetylated functional sperm proteins underexpressed in unilateral varicocele in comparison to bilateral varicocele.

Gene Symbol	Protein Name	Accession Number	Abundance		NSAF Ratio (UV vs. BV)
			UV	BV	
ACTB	actin, cytoplasmic 1	4501885	H	H	0.55
EEF1A1	elongation factor 1-alpha 1	4503471	H	H	0.56
FASN	fatty acid synthase	41872631	H	H	0.59
HSPA5	78 glucose-regulated protein precursor	16507237	H	H	0.56
HSP90AA1	heat shock protein HSP 90-alpha isoform 1	153792590	H	H	0.32
MYH9	myosin-9	12667788	H	H	0.4
PKM	pyruvate kinase isozymes M1/M2 isoform c	332164775	H	H	0.63
TUBA3C	tubulin alpha-3C/D chain	17921993	H	H	0.48
TUBB4B	tubulin beta-4B chain	5174735	H	H	0.41
ACAT1	acetyl-CoA acetyltransferase, mitochondrial precursor (involved in fatty acid metabolism and ketone body formation not lysine-acetylation of proteins)	4557237	L	H	0.19
P4HB	protein disulfide-isomerase precursor	20070125	L	H	0.31
ALDOA	fructose-bisphosphate aldolase A isoform 2	342187211	M	H	0.59
ANXA1	annexin A1	4502101	M	H	0.46
ANXA2	annexin A2 isoform 1	50845388	M	H	0.21
ANXA5	annexin A5	4502107	M	H	0.22
ANXA6	annexin A6 isoform 1	71773329	M	H	0.18
ATP5A1	ATP synthase subunit alpha, mitochondrial precursor	4757810	M	H	0.58
ATP5B	ATP synthase subunit beta, mitochondrial precursor	32189394	M	H	0.3
PDIA3	protein disulfide-isomerase A3 precursor	21361657	M	H	0.26
HK1	hexokinase-1 isoform HKI-ta/tb	15991831	M	H	0.5
HSPA8	heat shock cognate 71 protein isoform 1	5729877	M	H	0.37
HSP90AB1	heat shock protein HSP 90-beta	20149594	M	H	0.42
LCP1	plastin-2	167614506	M	H	0.65
HSP90B1	endoplasmin precursor	4507677	M	H	0.27
RUVBL1	ruvB-like 1	4506753	M	H	0.55
HYOU1	hypoxia up-regulated protein 1 precursor	5453832	M	H	0.17
CCT2	T-complex protein 1 subunit beta isoform 1	5453603	M	H	0.55
RUVBL2	ruvB-like 2	5730023	M	H	0.37
PHGDH	D-3-phosphoglycerate dehydrogenase	23308577	M	H	0.57
CANX	calnexin precursor	10716563	VL	H	0.08
AKR1B1	aldose reductase	4502049	VL	L	0.12
PSMD13	26S proteasome non-ATPase regulatory subunit 13 isoform 1	157502193	VL	L	0.05

UV: unilateral varicocele, BV: bilateral varicocele, H: high, M: moderate, L: low, VL: very low.

**Table 2:** Comparison of the enrichment of lysine-acetylated functional sperm proteins overexpressed in unilateral varicocele in comparison to bilateral varicocele.

Gene Symbol	Protein Name	Accession Number	Abundance		NSAF Ratio (UV vs. BV)
			UV	BV	
ENO1	alpha-enolase isoform 1	4503571	H	H	2.05
MDH2	malate dehydrogenase, mitochondrial precursor	21735621	H	H	3.91
ACADVL	very long-chain specific acyl-CoA dehydrogenase, mitochondrial isoform 2 precursor	76496475	H	L	10.53
PARK7	protein DJ-1	31543380	L	L	3.78
ACADM	medium-chain specific acyl-CoA dehydrogenase, mitochondrial isoform b precursor	187960098	M	L	6.83
ALDH9A1	4-trimethylaminobutyraldehyde dehydrogenase	115387104	M	L	12.28
CALM1	calmodulin	58218968	M	L	5.22
cpt2	carnitine O-palmitoyltransferase 2, mitochondrial precursor	4503023	M	L	2.24
HSPA9	stress-70 protein, mitochondrial precursor	24234688	M	L	2.29
IPO5	importin-5	24797086	M	L	4.17
PSMB1	proteasome subunit beta type-1	4506193	M	L	6.66
SOD2	superoxide dismutase [Mn], mitochondrial isoform A precursor	67782305	M	L	10.51
DNPEP	aspartyl aminopeptidase	156416028	M	L	5.45
HIST1H2AA	histone H2A type 1-A	25092737	M	L	3.86
HIST1H2BA	histone H2B type 1-A	24586679	M	L	2.40
DLD	dihydrolipoyl dehydrogenase, mitochondrial precursor	91199540	H	M	10.28
FLNB	filamin-B isoform 2	105990514	H	M	2.03
GPI	glucose-6-phosphate isomerase isoform 2	18201905	H	M	8.39
SERPINB6	serpin B6	41152086	H	M	4.68
LAP3	cytosol aminopeptidase	41393561	H	M	8.54
DECR1	2,4-dienoyl-CoA reductase, mitochondrial precursor	4503301	M	M	2.65
DLAT	dihydrolipoyllysine-residue acetyltransferase component of pyruvate dehydrogenase complex, mitochondrial precursor	31711992	M	M	2.36
DLST	dihydrolipoyllysine-residue succinyltransferase component of 2-oxoglutarate dehydrogenase complex, mitochondrial isoform 1 precursor	19923748	M	M	2.91
ETFA	electron transfer flavoprotein subunit alpha, mitochondrial isoform a	4503607	M	M	2.77
GSR	glutathione reductase, mitochondrial isoform 1 precursor	50301238	M	M	6.39
PRDX1	peroxiredoxin-1	320461711	M	M	2.04
SDHA	succinate dehydrogenase [ubiquinone] flavoprotein subunit, mitochondrial	156416003	M	M	2.35
APMAP	adipocyte plasma membrane-associated protein	24308201	M	M	2.45
ASRGL1	l-asparaginase	145275200	M	M	1.99
GOT2	aspartate aminotransferase, mitochondrial precursor	73486658	H	VL	31.16
CLGN	calmegin precursor	4758004	L	VL	3.77
FTH1	ferritin heavy chain	56682959	L	VL	6.51
HAGH	hydroxyacylglutathione hydrolase, mitochondrial isoform 1 precursor	94538322	L	VL	8.18
MDH1	malate dehydrogenase, cytoplasmic isoform 1	312283701	L	VL	5.46
MIF	macrophage migration inhibitory factor	4505185	L	VL	6.98
PPP3CA	serine/threonine-protein phosphatase 2B catalytic subunit alpha isoform isoform 2	194688147	L	VL	10.66
RPL5	60S ribosomal protein L5	14591909	L	VL	8.34
SUCLA2	succinyl-CoA ligase [ADP-forming] subunit beta, mitochondrial precursor	11321583	L	VL	46.32
LACTB2	beta-lactamase-like protein 2	7705793	L	VL	7.36
C1QBP	complement component 1 Q subcomponent-binding protein, mitochondrial precursor	4502491	M	VL	7.24
CAPN1	calpain-1 catalytic subunit	311893363	M	VL	5.51
FBP1	fructose-1,6-bisphosphatase 1	189083692	M	VL	26.30
SOD1	superoxide dismutase [Cu-Zn]	4507149	M	VL	5.68
TKT	transketolase	205277463	M	VL	11.17

UV: unilateral varicocele, BV: bilateral varicocele, H: high, M: moderate, L: low, VL: very low.

**Table 3:** Important proteins with enriched lysine acetylation uniquely expressed in the unilateral varicocele (UV) and bilateral varicocele (BV) group.

Gene Symbol	Protein Name	Accession Number	Abundance	Expression
GSS	glutathione synthetase	4504169	L	Unique in UV
PPP5C	serine/threonine-protein phosphatase 5 isoform 1	5453958	L	Unique in UV
TALDO1	transaldolase	5803187	L	Unique in UV
GNPDA1	glucosamine-6-phosphate isomerase 1	13027378	L	Unique in UV
CAT	catalase	4557014	M	Unique in UV
PNP	purine nucleoside phosphorylase	157168362	M	Unique in UV
SELENB P1	selenium-binding protein 1	16306550	M	Unique in UV
ACAA2	3-ketoacyl-CoA thiolase, mitochondrial	167614485	M	Unique in UV
ACTC1	actin, alpha cardiac muscle 1 proprotein	4885049	L	Unique in BV
ATP5C1	ATP synthase subunit gamma, mitochondrial isoform L (liver) precursor	50345988	L	Unique in BV
HMOX2	heme oxygenase 2	8051608	L	Unique in BV
PFN2	profilin-2 isoform a	16753215	L	Unique in BV
PHB	prohibitin	4505773	L	Unique in BV
VDAC1	voltage-dependent anion-selective channel protein 1	4507879	L	Unique in BV
YWHAB	14-3-3 protein beta/alpha	21328448	L	Unique in BV
YWHAG	14-3-3 protein gamma	21464101	L	Unique in BV
DNAJA2	dnaJ homolog subfamily A member 2	5031741	L	Unique in BV
STOML2	stomatin-like protein 2	7305503	L	Unique in BV
CYCS	cytochrome c	11128019	L	Unique in BV
MTPN	myotrophin	21956645	L	Unique in BV
7-Sep	septin-7 isoform 2	148352329	VL	Unique in BV
TAGLN2	transgelin-2	4507357	VL	Unique in BV

H: high, M: moderate, L: low, VL: very low.

**Table 4:** Potential acetylated protein biomarkers in the unilateral and bilateral varicocele group.

Sperm Function	DEPs	Unilateral Varicocele		Bilateral Varicocele	
		Abundance	Expression	Abundance	Expression
Fertilization and acrosome reaction	HIST1H2B	Moderate	OE	Low	UE
Mitochondrial dysfunction and oxidative stress	SDHA	Moderate	OE	Moderate	UE
	PRDX1	Moderate	OE	Moderate	UE
	SOD1	Moderate	OE	Very low	UE

DEPs: differentially expressed proteins, UE: underexpressed, OE: overexpressed.

## Supplementary Materials

The following are available online at https://www.mdpi.com/1422-0067/21/9/3155/s1, Supplementary Table S1. Semen parameters in infertile men with unilateral and bilateral varicocele. Supplementary Table S2. List of proteins involved in the acetylation process in the spermatozoa of unilateral and bilateral varicocele infertile men.

## References

[1] Samanta L., Durairajanayagam D. (2016). Introduction. Proteomics in Human Reproduction: Biomarkers for Millennials.

[2] Marmar J.L. (2001). The pathophysiology of varicoceles in the light of current molecular and genetic information. Hum. Reprod. Update.

[3] Dahl E.V., Herrick J.F. (1959). A vascular mechanism for maintaining testicular temperature by counter-current exchange. Surg. Gynecol. Obstet..

[4] Jarow J.P., Coburn M., Sigman M. (1996). Incidence of varicoceles in men with primary and secondary infertility. Urology.

[5] Baazeem A., Belzile E., Ciampi A., Dohle G., Jarvi K., Salonia A., Weidner W., Zini A. (2011). Varicocele and male factor infertility treatment: A new meta-analysis and review of the role of varicocele repair. Eur. Urol..

[6] Esteves S.C., Agarwal A. (2016). Afterword to varicocele and male infertility: Current concepts and future perspectives. Asian J. Androl..

[7] Hosseinifar H., Sabbaghian M., Nasrabadi D., Modarresi T., Dizaj A.V., Gourabi H., Gilani M.A. (2014). Study of the effect of varicocelectomy on sperm proteins expression in patients with varicocele and poor sperm quality by using two-dimensional gel electrophoresis. J. Assist. Reprod. Genet..

[8] Hosseinifar H., Gourabi H., Salekdeh G.H., Alikhani M., Mirshahvaladi S., Sabbaghian M., Modarresi T., Gilani M.A. (2013). Study of sperm protein profile in men with and without varicocele using two-dimensional gel electrophoresis. Urology.

[9] Camargo M., Intasqui Lopes P., Del Giudice P.T., Carvalho V.M., Cardozo K.H., Andreoni C., Fraietta R., Bertolla R.P. (2013). Unbiased label-free quantitative proteomic profiling and enriched proteomic pathways in seminal plasma of adult men before and after varicocelectomy. Hum. Reprod. (Oxford, England).

[10] Agarwal A., Sharma R., Samanta L., Durairajanayagam D., Sabanegh E. (2016). Proteomic signatures of infertile men with clinical varicocele and their validation studies reveal mitochondrial dysfunction leading to infertility. Asian J. Androl..

[11] Baker M.A., Nixon B., Naumovski N., Aitken R.J. (2012). Proteomic insights into the maturation and capacitation of mammalian spermatozoa. Syst. Biol. Reprod. Med..

[12] Yu H., Diao H., Wang C., Lin Y., Yu F., Lu H., Xu W., Li Z., Shi H., Zhao S. (2015). Acetylproteomic analysis reveals functional implications of lysine acetylation in human spermatozoa (sperm). Mol. Cell. Proteom. MCP.

[13] Krejci J., Stixova L., Pagacova E., Legartova S., Kozubek S., Lochmanova G., Zdrahal Z., Sehnalova P., Dabravolski S., Hejatko J. (2015). Post-Translational Modifications of Histones in Human Sperm. J. Cell. Biochem..

[14] Sun G., Jiang M., Zhou T., Guo Y., Cui Y., Guo X., Sha J. (2014). Insights into the lysine acetylproteome of human sperm. J. Proteom..

[15] Pang A., Rennert O. (2013). Protein acetylation and spermatogenesis. Reprod. Syst. Sex. Disord. Curr. Res..

[16] Agarwal A., Sharma R., Durairajanayagam D., Cui Z., Ayaz A., Gupta S., Willard B., Gopalan B., Sabanegh E. (2015). Differential proteomic profiling of spermatozoal proteins of infertile men with unilateral or bilateral varicocele. Urology.

[17] Samanta L., Swain N., Ayaz A., Venugopal V., Agarwal A. (2016). Post-Translational Modifications in sperm Proteome: The Chemistry of Proteome diversifications in the Pathophysiology of male factor infertility. Biochim. Biophys. Acta.

[18] Walker W.H., Easton E., Moreci R.S., Toocheck C., Anamthathmakula P., Jeyasuria P. (2015). Restoration of Spermatogenesis and Male Fertility Using an Androgen Receptor Transgene. PLoS ONE.

[19] O’Hara L., Smith L.B. (2015). Androgen receptor roles in spermatogenesis and infertility. Best Pract. Res. Clin. Endocrinol. Metab..

[20] Stanton P.G., Sluka P., Foo C.F., Stephens A.N., Smith A.I., McLachlan R.I., O’Donnell L. (2012). Proteomic changes in rat spermatogenesis in response to in vivo androgen manipulation; impact on meiotic cells. PLoS ONE.

[21] Solakidi S., Psarra A.M., Nikolaropoulos S., Sekeris C.E. (2005). Estrogen receptors alpha and beta (ERalpha and ERbeta) and androgen receptor (AR) in human sperm: Localization of ERbeta and AR in mitochondria of the midpiece. Hum. Reprod. (Oxford, England).

[22] Aquila S., Middea E., Catalano S., Marsico S., Lanzino M., Casaburi I., Barone I., Bruno R., Zupo S., Ando S. (2007). Human sperm express a functional androgen receptor: Effects on PI3K/AKT pathway. Hum. Reprod. (Oxford, England).

[23] Alkaram A., McCullough A. (2014). Varicocele and its effect on testosterone: Implications for the adolescent. Transl. Androl. Urol..

[24] Gioeli D., Paschal B.M. (2012). Post-translational modification of the androgen receptor. Mol. Cell. Endocrinol..

[25] Coffey K., Robson C.N. (2012). Regulation of the androgen receptor by post-translational modifications. J. Endocrinol..

[26] Riley T., Sontag E., Chen P., Levine A. (2008). Transcriptional control of human p53-regulated genes. Nat. Rev. Mol. Cell Biol..

[27] Raimondo S., Gentile T., Cuomo F., De Filippo S., Aprea G.E., Guida J. (2014). Quantitative evaluation of p53 as a new indicator of DNA damage in human spermatozoa. J. Hum. Reprod. Sci..

[28] Raimondo S., Gentile T., Gentile M., Morelli A., Donnarumma F., Cuomo F., De Filippo S., Montano L. (2019). p53 Protein Evaluation on Spermatozoa DNA in Fertile and Infertile Males. J. Hum. Reprod. Sci..

[29] Chang F.W., Sun G.H., Cheng Y.Y., Chen I.C., Chien H.H., Wu G.J. (2010). Effects of varicocele upon the expression of apoptosis-related proteins. Andrologia.

[30] Huang Y., Jin Y., Yan C.H., Yu Y., Bai J., Chen F., Zhao Y.Z., Fu S.B. (2008). Involvement of Annexin A2 in p53 induced apoptosis in lung cancer. Mol. Cell. Biochem..

[31] Munuce M.J., Marini P.E., Teijeiro J.M. (2019). Expression profile and distribution of Annexin A1, A2 and A5 in human semen. Andrologia.

[32] Nazmi A.R., Ozorowski G., Pejic M., Whitelegge J.P., Gerke V., Luecke H. (2012). N-terminal acetylation of annexin A2 is required for S100A10 binding. Biol. Chem..

[33] Ma Q. (2013). Role of nrf2 in oxidative stress and toxicity. Annu. Rev. Pharmacol. Toxicol..

[34] Dias T.R., Samanta L., Agarwal A., Pushparaj P.N., Panner Selvam M.K., Sharma R. (2019). Proteomic Signatures Reveal Differences in Stress Response, Antioxidant Defense and Proteasomal Activity in Fertile Men with High Seminal ROS Levels. Int. J. Mol. Sci..

[35] Samanta L., Agarwal A., Swain N., Sharma R., Gopalan B., Esteves S.C., Durairajanayagam D., Sabanegh E. (2018). Proteomic Signatures of Sperm Mitochondria in Varicocele: Clinical Use as Biomarkers of Varicocele Associated Infertility. J. Urol..

[36] Qiu X., Brown K., Hirschey M.D., Verdin E., Chen D. (2010). Calorie restriction reduces oxidative stress by SIRT3-mediated SOD2 activation. Cell Metab..

[37] Tao R., Coleman M.C., Pennington J.D., Ozden O., Park S.-H., Jiang H., Kim H.-S., Flynn C.R., Hill S., McDonald W.H. (2010). Sirt3-mediated deacetylation of evolutionarily conserved lysine 122 regulates MnSOD activity in response to stress. Mol. Cell.

[38] Chen Y., Zhang J., Lin Y., Lei Q., Guan K.L., Zhao S., Xiong Y. (2011). Tumour suppressor SIRT3 deacetylates and activates manganese superoxide dismutase to scavenge ROS. EMBO Rep..

[39] O’Flaherty C. (2014). Peroxiredoxins: Hidden players in the antioxidant defence of human spermatozoa. Basic Clin. Androl..

[40] Cui Z., Sharma R., Agarwal A. (2016). Proteomic analysis of mature and immature ejaculated spermatozoa from fertile men. Asian J. Androl..

[41] Parmigiani R.B., Xu W.S., Venta-Perez G., Erdjument-Bromage H., Yaneva M., Tempst P., Marks P.A. (2008). HDAC6 is a specific deacetylase of peroxiredoxins and is involved in redox regulation. Proc. Natl. Acad. Sci. USA.

[42] Swain N., Samanta L., Agarwal A., Kumar S., Dixit A., Gopalan B., Durairajanayagam D., Sharma R., Pushparaj P.N., Baskaran S. (2019). Aberrant Upregulation of Compensatory Redox Molecular Machines May Contribute to Sperm Dysfunction in Infertile Men with Unilateral Varicocele: A Proteomic Insight. Antioxid. Redox Signal..

[43] Nowicka-Bauer K., Lepczynski A., Ozgo M., Kamieniczna M., Fraczek M., Stanski L., Olszewska M., Malcher A., Skrzypczak W., Kurpisz M.K. (2018). Sperm mitochondrial dysfunction and oxidative stress as possible reasons for isolated asthenozoospermia. J. Physiol. Pharmacol. An Off. J. Pol. Physiol. Soc..

[44] Cimen H., Han M.J., Yang Y., Tong Q., Koc H., Koc E.C. (2010). Regulation of succinate dehydrogenase activity by SIRT3 in mammalian mitochondria. Biochemistry.

[45] Finley L.W., Haas W., Desquiret-Dumas V., Wallace D.C., Procaccio V., Gygi S.P., Haigis M.C. (2011). Succinate dehydrogenase is a direct target of sirtuin 3 deacetylase activity. PLoS ONE.

[46] Wang D., Fang C., Zong N.C., Liem D.A., Cadeiras M., Scruggs S.B., Yu H., Kim A.K., Yang P., Deng M. (2013). Regulation of acetylation restores proteolytic function of diseased myocardium in mouse and human. Mol. Cell. Proteom. MCP.

[47] Kerns K., Morales P., Sutovsky P. (2016). Regulation of sperm capacitation by the 26S proteasome: An emerging new paradigm in spermatology. Biol. Reprod..

[48] Zigo M., Manaskova-Postlerova P., Jonakova V., Kerns K., Sutovsky P. (2019). Compartmentalization of the proteasome-interacting proteins during sperm capacitation. Sci. Rep..

[49] Schwartz D., Gygi S.P. (2005). An iterative statistical approach to the identification of protein phosphorylation motifs from large-scale data sets. Nat. Biotechnol..

[50] Kramer A., Green J., Pollard J., Tugendreich S. (2014). Causal analysis approaches in Ingenuity Pathway Analysis. Bioinformatics (Oxford, England).

[51] Narushima Y., Kozuka-Hata H., Koyama-Nasu R., Tsumoto K., Inoue J., Akiyama T., Oyama M. (2016). Integrative Network Analysis Combined with Quantitative Phosphoproteomics Reveals Transforming Growth Factor-beta Receptor type-2 (TGFBR2) as a Novel Regulator of Glioblastoma Stem Cell Properties. Mol. Cell. Proteom. MCP.

[52] Panner Selvam M.K., Agarwal A., Dias T.R., Martins A.D., Samanta L. (2019). Presence of Round Cells Proteins do not Interfere with Identification of Human Sperm Proteins from Frozen Semen Samples by LC-MS/MS. Int. J. Mol. Sci..

