# Supplementary figures and images for: Functional Analysis of Differentially Expressed Acetylated Spermatozoal Proteins in Infertile Men with Unilateral and Bilateral Varicocele
Source: Int J Mol Sci. 2020 Apr 30;21(9):3155. doi: 10.3390/ijms21093155 (PMC7246524; doi:10.3390/ijms21093155)

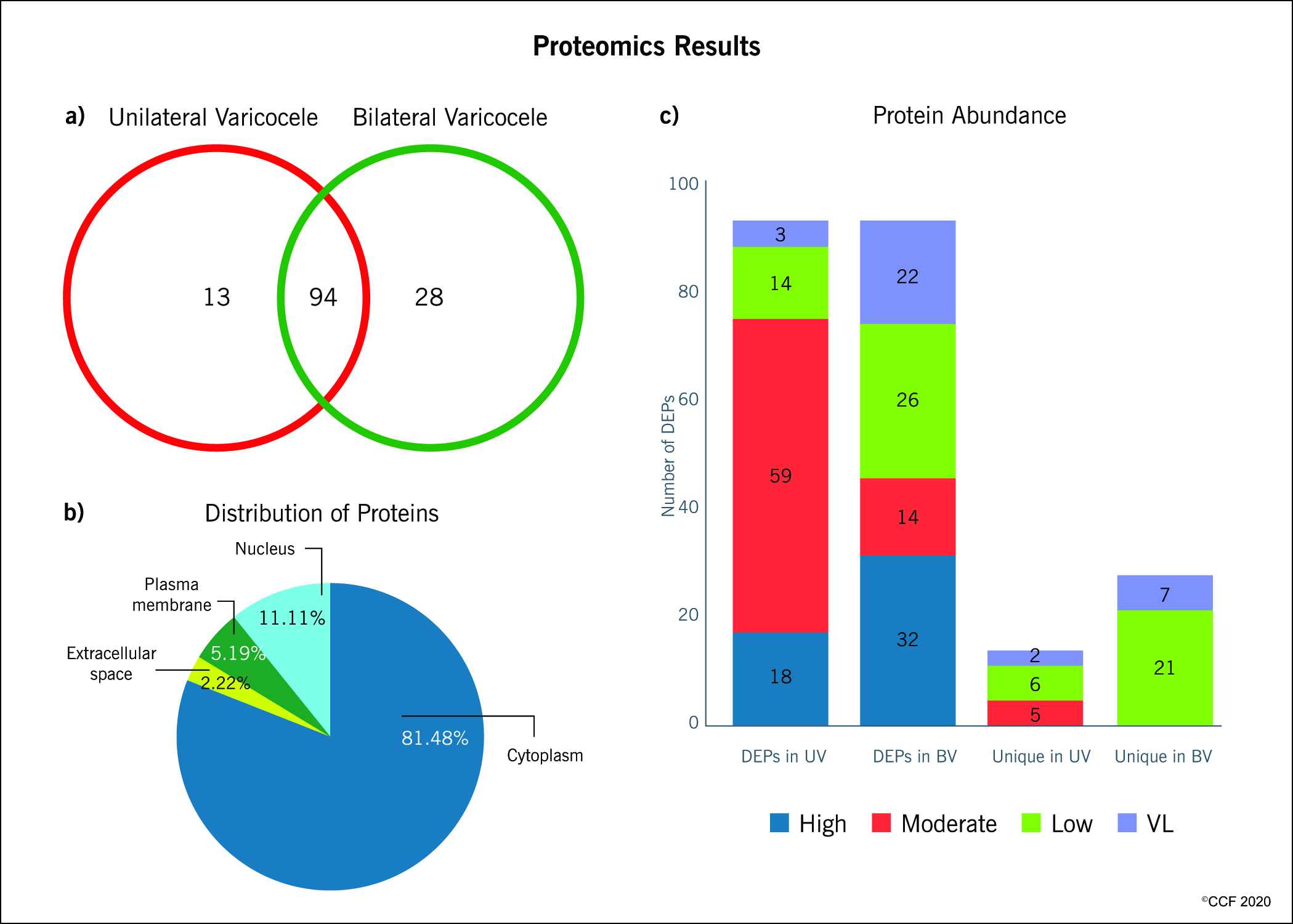

Supplement: Supplementary file 1 [file ijms-21-03155-s001.zip › Figures/Figure 1.tif]

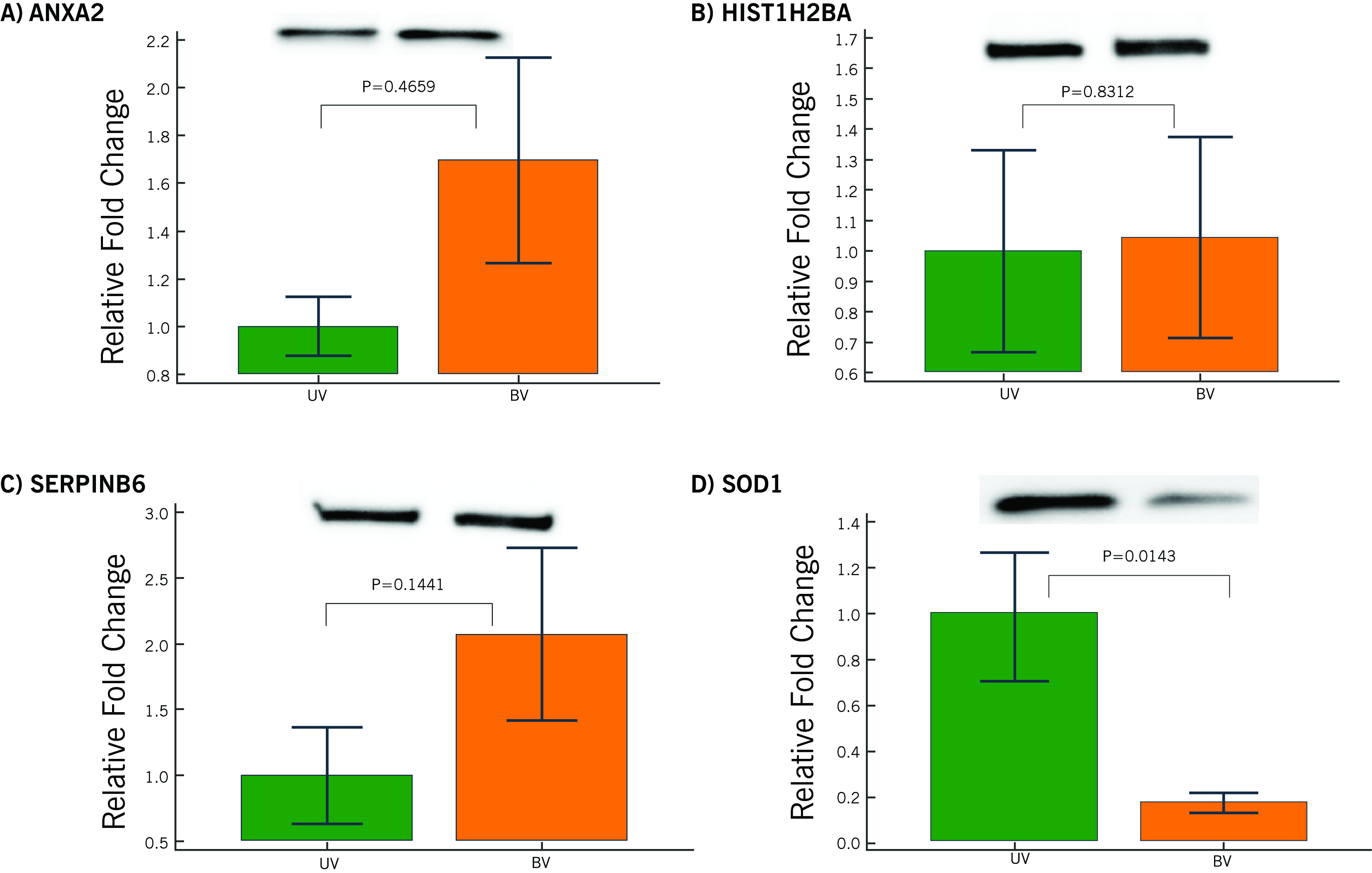

Supplement: Supplementary file 1 [file ijms-21-03155-s001.zip › Figures/Figure 2.tif]
